# Crosstalk between thyroid CSCs and immune cells: basic principles and clinical implications

**DOI:** 10.3389/fimmu.2024.1476427

**Published:** 2024-12-24

**Authors:** Xiaoxiao Li, Hengtong Han, Kaili Yang, Shouhua Li, Libin Ma, Ze Yang, Yong-xun Zhao

**Affiliations:** ^1^ The First School of Clinical Medicine, Lanzhou University, Lanzhou, China; ^2^ The Seventh Department of General Surgery, Department of Thyroid Surgery, The First Hospital of Lanzhou University, Lanzhou, China

**Keywords:** thyroid cancer stem cells, thyroid cancer, immune cells, targeting cancer stem cells, immunotherapy

## Abstract

Thyroid cancer has become the most common endocrine malignancy. Although the majority of differentiated thyroid cancers have a favorable prognosis, advanced thyroid cancers, iodine-refractory thyroid cancers, and highly malignant undifferentiated carcinomas still face a serious challenge of poor prognosis and even death. Cancer stem cells are recognized as one of the central drivers of tumor evolution, recurrence and treatment resistance. A fresh viewpoint on the oncological aspects of thyroid cancer, including proliferation, invasion, recurrence, metastasis, and therapeutic resistance, has been made possible by the recent thorough understanding of the defining and developing features as well as the plasticity of thyroid cancer stem cells (TCSCs). The above characteristics of TCSCs are complicated and regulated by cell-intrinsic mechanisms (including activation of key stem signaling pathways, somatic cell dedifferentiation, etc.) and cell-extrinsic mechanisms. The complex communication between TCSCs and the infiltrating immune cell populations in the tumor microenvironment (TME) is a paradigm for cell-extrinsic regulators. This review introduces the current advances in the studies of TCSCs, including the origin of TCSCs, the intrinsic signaling pathways regulating the stemness of TCSCs, and emerging biomarkers; We further highlight the underlying principles of bidirectional crosstalk between TCSCs and immune cell populations driving thyroid cancer progression, recurrence, or metastasis, including the specific mechanisms by which immune cells maintain the stemness and other properties of TCSCs and how TCSCs reshape the immune microenvironmental landscape to create an immune evasive and pro-tumorigenic ecological niche. Finally, we outline promising strategies and challenges for targeting key programs in the TCSCs-immune cell crosstalk process to treat thyroid cancer.

## 1 Introduction

Thyroid cancer (TC) is the most prevalent endocrine cancer (1). According to the latest GLOBOCAN data released by THE INTERNATIONAL AGENCY, thyroid cancer has become the seventh most prevalent cancer worldwide (2). The American Association statistics show a dramatic increase in TC incidence from 2000, with 44,020 new cases and 2,170 TC deaths expected in the United States by 2024 (3). Histologically, thyroid cancers are mainly classified into differentiated thyroid carcinoma (DTC) originating from follicular cells, undifferentiated thyroid carcinoma (ATC), and medullary carcinoma (MTC) originating from parafollicular C-cells. Of these, differentiated thyroid carcinoma (DTC), among which DTC with a favorable prognosis accounts for 95% of cases (4). Multimodal therapeutic strategies such as surgery, chemotherapy, radioactive iodine (RAI), immunotherapy, and targeting of disease-causing genes have demonstrated significant extension of overall survival (OS) and progression-free survival (PFS), and even cure the majority of DTC patients (5). Even though targeted medicines and immunotherapies show some success, a small percentage of patients with advanced DTC and the majority of patients with ATC and MTC eventually develop acquired resistance to them. Furthermore, even individuals who are in a state of remission following treatment remain at risk of relapse and subsequent metastasis in the short term. These phenomena are closely linked to the biology of cancer stem cells (CSCs).

Cancer stem cells (CSCs) represent a subset of cancer cells endowed with properties such as self-renewal, differentiation into various kinds of cancerous cells, resistance to conventional treatments, and significant migratory capabilities (6). Previously, CSCs were considered to be the “Hierarchical organization theory” of the apex population with an infinite capacity for self-proliferation, which promotes and sustains the growth and spread of tumors (7). However, the latest dynamic CSCs model emphasizes that they are differentiated states of cells under plasticity, that is, cancer cells that are already highly differentiated exhibit a remarkable ability to regress to a more primitive or de-differentiated state under specific environments or stimuli (8). Using a flow cytometric analysis technique, Mitsutake et al. (9) discovered that thyroid cancer cells have significant tumorigenicity, differentiation plasticity, and phenotypic heterogeneity as early as 2007, confirming the presence of thyroid cancer stem cells (TCSCs) in TC. Recent studies have shown that TCSCs are key drivers of proliferation, invasion, metastasis, recurrence, and treatment resistance in some thyroid cancer patients (10). Indeed, the tumorigenic biology exhibited by TCSCs involves the complex regulation of multiple factors both inside and external to cells (11). In the first instance, Under the control of intrinsic drivers, such as the overexpression of specific pluripotent transcription factors (like OCT4, Nanog, and Sox2) or aberrant activation of stemness-associated pathways (like Wnt, Notch, Hedgehog, and other signaling pathways), TCSCs drive the process of tumorigenesis and progression (12, 13); On the other hand, there are exogenous cues in the tumor microenvironment (TME) that sustain, bolster, or impede the fate of TCSCs. Particularly, a complex series of exchanges between TCSCs and immune cells in the TME through an array of modalities such as the release of soluble factors, metabolic reprogramming products, exosomes, and receptor-ligand binding influence, to some extent, the presentation of stemness and plasticity of TCSCs, and even have far-reaching impacts on the formation and evolution of the TME’s Immune landscape (14). However, the precise regulatory mechanisms underlying TCSCs, as well as their plasticity, stemness, and other biological characteristics, as well as their role in TC pathogenesis, progression, metastasis, and recurrence, are still mostly unknown; moreover, the detailed landscape of the interactions and exchanges between TCSCs and the key components in the TME still needs to be further explored.

This paper describes current advances in the study of TCSCs, including the origins of TCSCs, specific biomarkers, and the intrinsic signaling pathways that regulate the important properties of TCSCs. In addition, this paper focuses on the mechanisms by which bidirectional interaction between TCSCs and the immune system drives TC development, and evaluates the potential to develop more precise and personalized antitumor therapies by targeting key targets or events in the TCSCs-immune cell crosstalk process. Finally, we summarize the pending problems and solutions in this field.

## 2 Overview of TCSCs

### 2.1 Origin of TCSCs

Studies related to tumor biology have revealed extensive intra-tumor heterogeneity (15), that is, cancerous tissue includes cancer cells in a variety of stages of differentiation. These cancer cells differ in gene expression, immune interactions, tumor proliferation potential, and response to treatment. Explanations for these differences are varied, including different genetic and epigenetic changes as well as the microenvironmental features of the tumor.

**Figure 1:**
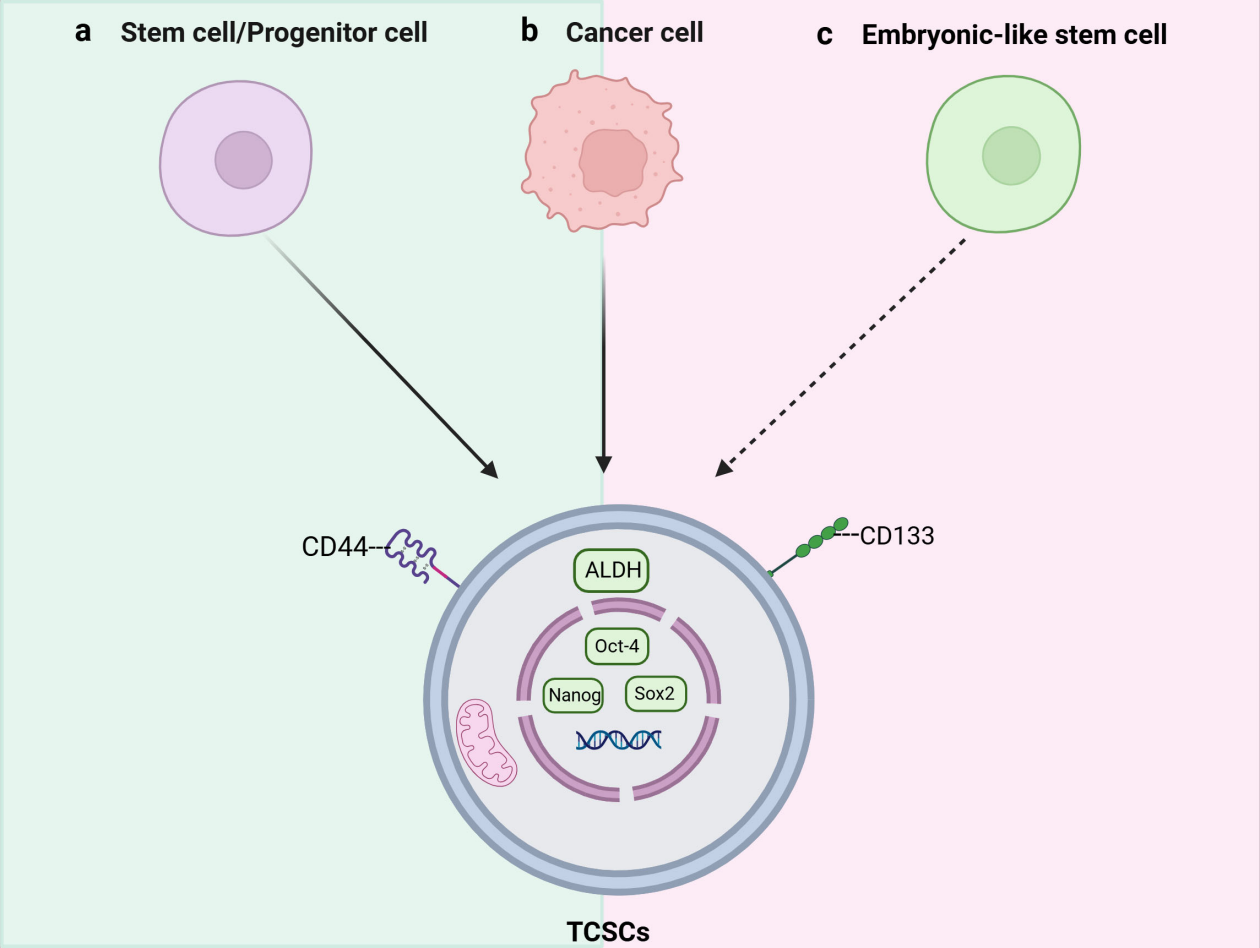
Schematic representation of the origins and key markers of tumor stem cells (TCSCs). TCSCs can arise from **(A)** stem cells/progenitor cells, **(B)** Cancer cells, and **(C)** Embryonic-like stem cells.

An increasing amount of data points to TCSCs as a key model for explaining TC intratumor heterogeneity (16). Mitsutake et al. initially revealed in 2007 that TC samples contained a side population (SP) of highly enriched thyroid cancer stem cells that could both self-renew and create non-SP cells by asymmetric divisions and tumor formation in naked mice (9). While the presence of TCSCs has been widely accepted, there is ongoing debate regarding the cell source. Initially, Zhang et al. (10) suggested that TCs require multistep mutations for TC development, and thus it is more likely that TCSCs originate from self-renewing stem/progenitor cells that alter only the proliferative pathway, rather than from mature cells with a short lifespan. For example, rearrangements of RET/PTC in papillary thyroid carcinoma are more likely to lead to sustained cell proliferation and cancer development when they occur in thyroid stem cells (17). However, there is growing evidence that they can also originate from dedifferentiated thyroid cancer cells. For example, Ma et al. (18), knocked the BRAFV600E gene specifically into the mouse thyroid, and found a decrease in the expression of thyroid-specific genes (such as Tg and NIS) as well as a significant increase in the expression of stemness markers (e.g., Oct4, Rex1, CD15, and Sox2); what is more, this study conclusively demonstrates that thyroid epithelial cells possess the potential to transform into stem cell-like cells, which expands the new field of cellular plasticity research. Thus, the stemness of thyroid cancer is at least partially acquired by the epithelial-mesenchymal transition (EMT) process undergone by thyroid cancer cells. Gil et al. (19) also suggested that embryonic stem cell-like cells in blood or other tissues can be another origin of CSCs after transformation under inappropriate conditions. However, whether the above-mentioned CSCs origin pattern exists in thyroid cancer needs to be further verified. It is not clear what kind of cells TCSCs originate from, but stem/progenitor cells and cancer cells may become the origin of TCSCs through “transformation” and “dedifferentiation”, respectively (10, 20) (**Figure 1**). In addition, it is important that the aforementioned diverse starting cells may individually show distinct roles and effects in different thyroid cancer subtypes or at various phases of the disease’s course.

### 2.2 Stemness markers

Not only can TCSCs-specific stemness markers be utilized to distinguish and separate TCSCs from TC, they may also determine the internal diversity of TCSCs, and more importantly, participate in signal transduction and cell-cell interactions as well. Therefore, we summarize and discuss specific cell surface biomarkers and pluripotent transcription factors of TCSCs and their potential roles in TCSCs regulation (**Table 1**), which will assist in identifying and isolating thyroid cancer stem cells as well as creating possible treatment plans that specifically target these cells.

**Table 1:** Markers of thyroid cancer stem cells.

Marker	Characteristic	Function in Thyroid Cancer	References
CD133	five-transmembrane glycoprotein	Linked to more aggressive forms of thyroid cancer and high tumorigenicity, aggression, and resistance to therapy	(21)
CD44	glycosylated transmembrane glycoprotein	TC is associated with tumorigenesis, progression, treatment resistance and poor prognosis; associated with BRAF mutations; regulates intracellular signaling	(22–26)
ALDH	aldehyde dehydrogenase	Self-renewal and linked to the expansion of tumors;	(16)
OCT-4	transcription factor	Tumorigenesis, EMT Induction, and Treatment Resistance Correlation	(27–29)
Nanog	homeodomain protein	Responsible for preserving TCSCs pluripotency and controlling their ability to proliferate and self-renew; greater propensity for tumor start	(30, 31)
SOX2	transcription factor	Involved in regulating TCSCs self-renewal and tumor growth; in addition, SOX2 has been implicated in treatment resistance	(32, 33)
CHD4	ATPase core subunit	Correlates with stemness and self-renewal of TCSCs	(34)
SSEA-1(CD15)	Carbohydrate epitope	A human embryonic stem cell early differentiation marker that differentially expresses high levels of stem cell-associated genes in human thyroid cancer cell lines, suggesting that SSEA-1 can be used as a marker for TCSCs.	(18)

CD133 is a highly conserved antigenic homologue of mouse Prominin-1 pentane transmembrane glycoprotein. It is not only an important biomarker for recognizing and isolating TCSCs, but also plays a closely related role in self-renewal, tumorigenicity, invasiveness, and therapeutic resistance of TCSCs (35). For example, Friedman et al. (21) showed for the first time that injecting CD133+ cells into immunodeficient NOD/SCID mice results in the creation of tumors. In addition, CD133 has been associated with aggressive thyroid cancers; for example, Xu et al. (36) used immunohistochemistry to find that CD133 activated the Akt signaling pathway in thyroid tumors that were either poorly differentiated or undifferentiated, increasing the TCSCs’ capacity for self-renewal and tumorigenesis (37), and most recent research, Wang et al. (38) discovered that CD133 could also significantly increase the transcriptional level of the glutamate-aspartate transporter SLC1A3 in CD133+ thyroid cancer cells by triggering the NF-κB signaling pathway. Further evidence suggests that CD133 is also associated with treatment resistance. When MTC cell lines were treated with 5-fluorouracil (5-FU), it was observed that the CD133+ tumor-originating subpopulation exhibited significantly enhanced resistance to chemotherapeutic agents and that this resistance to chemotherapeutic agents was stably maintained over a long period of time, revealing the potential challenge of this subpopulation in tumor therapy (39).

CD44 is a common marker in the identification of TCSCs, and binding of CD44 to hyaluronic acid (an important glycosaminoglycan, or HA) in the components of the extracellular matrix (ECM) has been reported to correlate with TCSCs migration and invasiveness (22–24, 40). It has been shown that CD44 is also associated with TC progression, for example, PTC tissue samples with a combination of CD44 and CD24+/- were associated with extra-thyroidal metastases and negatively correlated with recurrence-free survival (RFS) (25). In addition, Within TCSCs, CD44 plays a role in controlling signaling pathways, such as activation of CREB transcription factor and upregulation of cell cycle protein D1 expression, which promotes the proliferation of TCSCs (26). These studies indicate that CD44 plays a significant role in promoting TC formation as well as metastasis and has been identified as a possible therapeutic target.

It is commonly acknowledged that the core transcription factor Octamer-binding protein 4 (OCT-4) controls the allosteric potential and self-renewal capacity of CSCs (30). Research has confirmed that OCT-4 is overexpressed in TCSCs and is closely linked to thyroid tumorigenesis, induction of the EMT process, and treatment resistance (27–29, 41). For example, thyroid mesenchymal carcinoma cells carrying the stem cell markers NANOG and Oct4 were able to initiate tumor growth after transplantation into immunodeficient mice (27).

### 2.3 Important signaling pathways that control TCSCs characteristics

**Figure 2:**
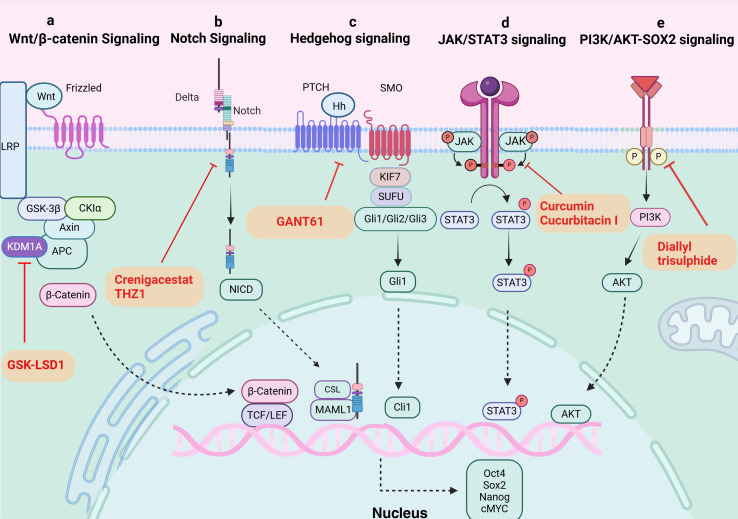
Signaling pathways in TCSCs. **(A)** Wnt/β-catenin signaling pathway. KDM1A demethylates APC2 and DKK1, triggers Wnt signaling, starts the transcription of downstream target genes, and raises expression of stemness marker proteins. GSK-LSD1 effectively blocked KDM1A’s demethylation, preventing the abnormal stimulation of the Wnt/β-catenin pathway. **(B)** Notch signaling pathway. The ligand attaches itself to the Notch extracellular receptor, releasing the Notch intracellular structural domain (NICD). This NICD enters the nucleus and interacts with the CSL to control the expression of target genes downstream. Crenigacestat efficiently prevents TCSCs-driven tumor growth *in vivo* by reducing aldehyde dehydrogenase activity and blocking the Notch1-cMYC signaling pathway, which prevents the development of tumor balls. **(C)** Hippo signaling pathway. The Hh ligand attaches to the extracellular structural domain of PTCH upon activation of Hh signaling, blocking the receptor and removing its inhibitory action on SMO. Gli proteins can be dephosphorylated and transformed into their active state when Sufu’s function is inhibited by Smo. Target genes (like Snail) are expressed more when activated Gli1 enters the nucleus. By inhibiting Gli1, GANT61 suppresses the Shh pathway, delaying the formation of tumors fueled by TCSCs. **(D)** JAK/STAT signaling pathway. The cytokines control target gene transcription by binding to membrane receptors, activating JAKs, and mediating STAT phosphorylation into the nucleus. Through their targeting of the JAK/STAT3 signaling pathway, curcumin and cucurbitacin suppressed the expression of stemness genes and the tumor-promoting potential of TCSCs. **(E)** PI3K/AKT-SOX2 signaling. Diallyl trisulphide inhibited the tumorigenic ability of TCSCs by inhibiting the PI3K/AKT pathway, reducing the phosphorylation level of AKT and downstream SOX2 expression.

Several critical signaling pathways that are necessary for the survival, proliferation, self-renewal, and differentiation of normal stem cells were shown to be aberrantly activated in the mechanism of TCSCs-induced cancer. Typical examples include the Wnt/β-catenin, Sonic Hedgehog (Shh), and Notch signaling pathways (42) (**Figure 2**), which intertwine to form a complex network that together determine the stemness and multidirectional differentiation potential of TCSCs.

The classical Wnt/β-catenin signaling pathway plays a major role in many complex physiological activities *in vivo*, including cell development, proliferation, differentiation, apoptosis, migration, invasion, and tissue homeostasis (43, 44). And The Wnt/β-catenin signaling pathway’s aberrant activation state exerts certain effects on the self-renewal and proliferation of TCSCs. For instance, β-catenin nuclear translocation in TCSCs (45), enhances sphere formation and resistance to 131I treatment. In addition, new mechanisms to activate the pathway have been recently discovered, and lysine-specific histone demethylase 1A (KDM1A), an important epigenetic modifier, has been closely associated with Wnt/β-catenin pathway activation (46). KDM1A’s demethylation activity affected both the APC2 promoter area and H3K4me1/2 on HIF-1α, which led to the downregulation of DKK1 and APC2 expression (46). In addition to blocking APC2 transcription, this also triggered the HIF-2α/miR-146a/DKK1 axis, which subsequently facilitated the Wnt signaling pathway’s activation, boosted the expression of stemness marker proteins, and enhanced the sphere formation and chemoresistance of thyroid cancer cells (46). Furthermore, it’s possible that the oncogene DAPK1 (death-associated protein kinase 1) controls β-catenin negatively. The inhibition of DAPK1 expression resulted in a considerable activation of the Wnt/β-catenin signaling pathway. This alteration was accompanied by the elevation of markers characteristic to stem cells, such as Oct4, Sox2, and Nanog, as well as an enhancement of the ability for sphere formation (47).

The Notch family consists of Notch-1-4, whose activation process effectively inhibits programmed cell death (apoptosis), a mechanism that has been shown in multiple studies, and promotes cell viability, self-renewal mechanisms, and migratory behaviors (48, 49). Li et al. (50) treated the NOTCH1 inhibitor, crenigacestat, or Cyclin-dependent kinase 7 (CDK7) inhibitors with TCSCs, successfully inhibited the activity of TCSCs and hindered the initiation and growth of ATC cell-induced tumors *in vivo*. The above findings shed light on NOTCH1’s significance as an essential regulator of TCSCs activity and further demonstrate the critical role that NOTCH1 and its downstream signaling molecule, cMYC, play in preserving the distinctive characteristics of TCSCs. These discoveries open up new avenues for understanding the biological behavior of TCSCs (50).

The hedgehog (Hh) signaling pathway promotes self-renewal of TCSCs and enhances chemoresistance, and is a key signaling pathway that maintains TCSCs stemness and drives tumor growth, metastasis, and recurrence (51, 52). For example, by inhibiting the Shh pathway or reducing the amount of Gli1, a downstream effector of Shh signaling, Snail expression was found to be decreased and significantly reduced the number of TCSCs, demonstrating that the Shh pathway activates the transcriptional regulator Gli1, which in turn controls the production of Snail protein, preserving the stemness properties of TCSCs and encouraging their self-renewal (51). Furthermore, it has also been shown that the Shh signaling pathway can affect the phosphorylation status of AKT and c-Met in TCSCs through the regulation of Gli1, which thus encourages TCSCs mobility and invasion potential (52).

Taken together, the role of activation of aberrant signaling pathways in maintaining the stemness (e.g., self-renewal and sphere-forming ability) and multispectral differentiation potential of TCSCs is clear, and thus targeting aberrant signaling pathways can help eradicate TCSCs and improve the clinical efficacy and long-term prognosis of thyroid cancer patients (32). More notably, TCSCs are not only regulated internally, but they are also significantly and deeply shaped by their cell-external tumor microenvironment on both a structural and functional level. Therefore, in order to propose more effective intervention options, it is imperative to further identify the tumor microenvironmental signals that support the survival and development of TCSCs.

## 3 Crosstalk between TCSCs and immune cells

### 3.1 Ecological niche of TCSCs

**Figure 3:**
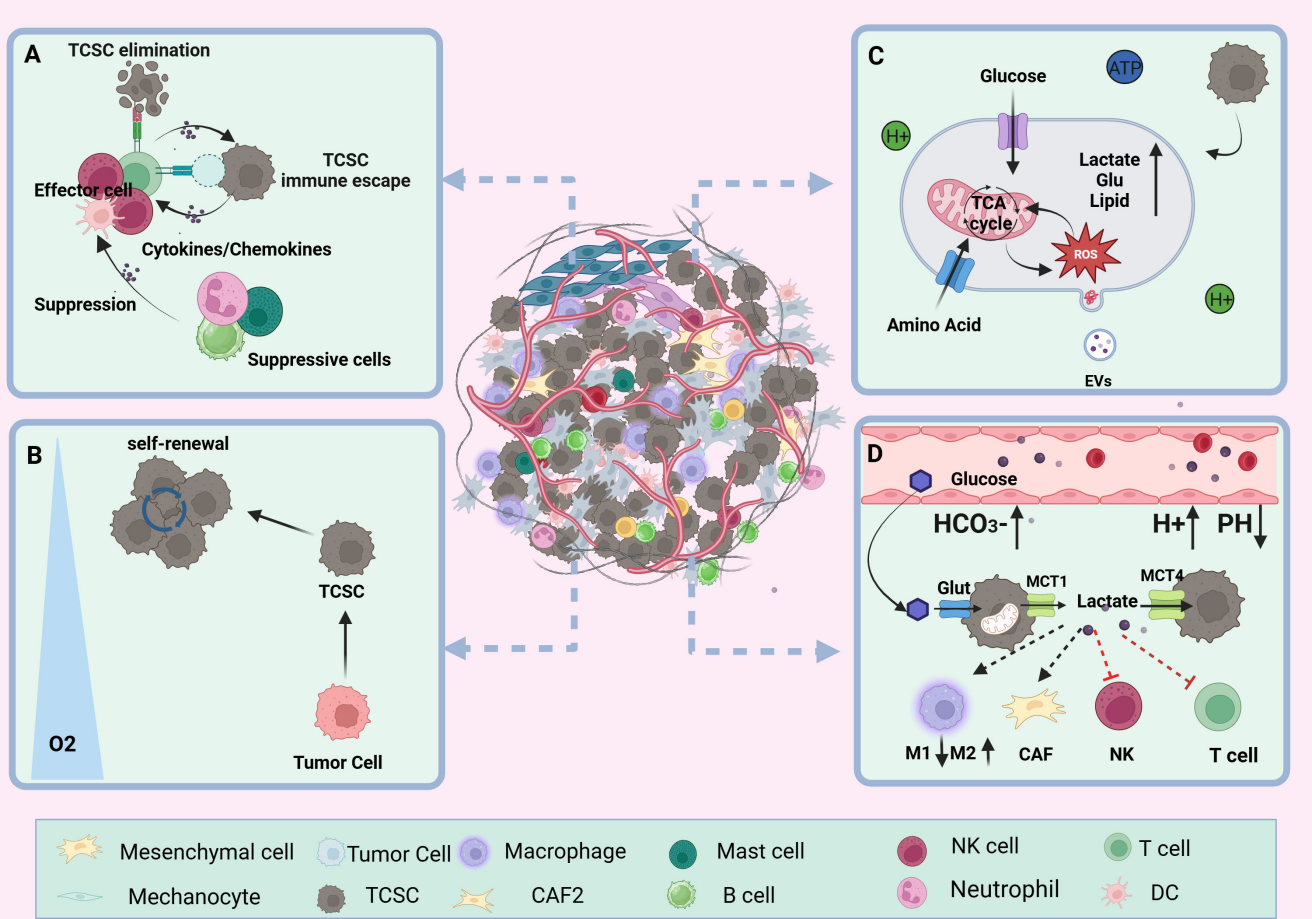
TCSCs ecological niche. **(A)** immune microenvironment. Through paracrine signaling or direct cell-to-cell contact, TCSCs communicate with immune cell cells in a variety of complex ways. This interaction influences the TME immune landscape’s formation and evolution and, to some extent, shapes the TCSCs’ capacity for self-renewal and multidirectional differentiation. **(B)** hypoxic microenvironment. Rapid tumor growth, pathologic angiogenesis, and stromal fibrosis all contribute to the TC’s inadequate oxygen supply in some areas. HIF-1α mediates hypoxia to suppress anti-tumor immunity and enhance the dryness of cancer cells, promoting the stem cell-like properties of thyroid cancer. **(C)** metabolic microenvironment. Through metabolic reprogramming, TCSCs effectively use scarce nutritional resources, such as glucose and glutamine, to satisfy the need for fast multiplication. However, the metabolic wastes they generate, including lactate and ROS, can also impair immune cell function. **(D)** acidic microenvironment. TCSCs accelerate the advancement of TC by increasing the production of lactic acid and the glycolytic pathway. This, in conjunction with protons and carbonic acid, creates an acidic milieu that supports TCSCs survival and impair immune cell function and provide an immunosuppressive environment that promotes the growth of tumors.

The TME is an extremely intricate and constantly shifting ecosystem made up primarily of the ECM and different types of cells (including cancer fibroblasts, vascular endothelial cells, immune cells, tumor cells, and other cells), and a variety of signaling molecules that sustain the TME’s connections, create immunosuppressive networks, undermine tumor-specific immunity, and effectively stimulate tumor growth (53, 54) (**Figure 3**). Researchers have become more interested in the niche in which cancer stem cells (CSCs) live due to the amazing success and enormous potential of immunotherapy in the treatment of solid tumors and hematologic malignancies (55). Ecological niche help TC development by preserving the phenotypic plasticity of TCSCs, increasing their survival, and shielding them from immune cell attacks (56).

#### 3.1.1 Immune microenvironment

TCSCs are well adapted to the dynamics of the TME and engage in complex and diverse exchanges with immune cells via paracrine signals or direct cell-to-cell contact, which to some extent shapes the self-renewal and multidirectional differentiation properties of TCSCs and even exerts a significant influence on the formation and evolution of the TME immune landscape (6). For example, TCSCs secrete exosomes loaded with CDKN2B-AS1, in thyroid cancer cells, which aberrantly activate the TGF-β1/Smad2/3 signaling pathway thereby promoting further tumor progression (57). In addition, TCSCs have the ability to express high quantities of programmed death ligand 1 (PD-L1), which trigger an ineffective anti-tumor immune response and promotes the development of an immunosuppressive surroundings by linking to the cognate receptor programmed death 1 (PD-1) on T cells (14). Conversely, immune cells recruited into the TME release an assortment of cytokines such as IL-6, IL-8, and other immunosuppressive factors and TGF-β; they upregulate the levels of the mesenchymal marker vimentin and drive the activation of TCSCs stemness-associated pathways (e.g., Akt-Slug) and stemness genes (e.g., OCT4 and ABCG2), which in turn enhances TCSCs self -renewal and TC invasiveness (56, 58, 59). For example, IL-8 produced by a range of immunological cells (such as neutrophils, macrophages, mast cells, etc.) and TCSCs, which specifically bind to CXCR1 and CXCR2, not only promote TCSCs’ self-renewal, sphere formation, and tumor-initiating ability through autocrine circuits but also maintains TCSCs stemness traits through paracrine Akt-Slug-dependent pathways and tumor initiation capacity (60–63). Furthermore, it was found that CXCR4-expressing TCSCs follow a chemokine gradient binding to stromal cell-derived factor-1 (SDF-1/CXCL12) released by cancer-associated fibroblasts (CAFs), which increases TCSCs invasiveness and promotes TC metastasis (64, 65).

#### 3.1.2 Hypoxic microenvironment

Compared with adjacent normal thyroid tissue, TC is mainly characterized by areas of Hypoxia inducible factor-1 (HIF-1α)-mediated hypoxia and has a wide range of implications for malignant progression and poor outlook of thyroid cancer (66). The hypoxic microenvironment is mainly due to stromal fibrosis, rapid tumor proliferation, and pathological angiogenesis, resulting in an imbalance between oxygen consumption and oxygen supply (67). Zhao et al. used CIBERSORT analysis to show that HIF1A was negatively correlated with the number and functional state of anti-tumor immune-related CD8+ T cells, NK cells, and monocytes in TCs under hypoxic conditions. On the other hand, it was positively correlated with dendritic cell activation, which enhances the immune response to tumors, and with T cell regulatory function (68). Notably, apart from inhibiting the immune system and encouraging the aggressiveness of thyroid tumors, hypoxia is also a potential factor regulating the fate of TCSCs. For example, the “stemness” property of thyroid cancer cells is closely related to the hypoxic microenvironment in which they are exposed, and Mankamova et al. (69) showed that hypoxia, which was mediated by HIF-1alpha, elevated the expression of mRNAs for stem cell markers (Oct4, Sox2, and Nanog) and increased the number of SPs, suggesting that hypoxic conditions greatly contribute to the stem cell-like properties of cancer cells.

#### 3.1.3 Metabolic microenvironment

Cancer's metabolic reprogramming is one of the known characteristics of tumor growth. Tumor cells regulate metabolic pathways, which include the abnormal production of signaling molecules and metabolism-related proteins, in response to the high metabolic needs of their own fast proliferation. Through trophic competition and metabolites, TCSCs modify the TME, leading immune cells to undergo metabolic reprogramming, significantly changing immune cell function, and encouraging tumor cell immune escape (70, 71). Wang et al. (37) found that through STAT3 phosphorylation, CD133+ thyroid cancer cells upregulate the expression of the NADPH oxidase 1 (NOX1) promoter, which contributes to the generation of reactive oxygen species (ROS). Following this, TCSCs’ Akt signaling is activated, increasing the generation of ROS to support tumor sphere formation and preserve self-renewal capabilities. According to Wang et al. (38) thyroid cancer cells with CD133+ had a greater glutamate level than thyroid cancer cells with CD133- and they further suggested that this phenomenon was mediated by the activation of NF-κB signaling to promote the transcriptional levels of glutamate aspartate transporter (SLC1A3), which in turn conferred TCSCs a stronger self-renewal ability. In addition to facilitating the ongoing development and survival of TCSCs, elevated glutamate levels in the TME advance the course of TC by diminishing neutrophil cytotoxicity and impeding T cell proliferation (38, 72).

#### 3.1.4 Acidic microenvironment

Cancer cells are prone to metabolize glucose through the glycolysis pathway even when oxygen is abundant. This metabolic pathway produces ATP more rapidly and generates a large amount of lactic acid, which is known as the “Warburg effect” (73). Subsequently, lactate is released into the TME via transport proteins such as MCT1 and MCT4 (74). The accumulation of lactate and the presence of other factors, such as protons (H+) and carbonic acid, combine to shape an acidic TME more favorable for tumor endurance and proliferation in an assortment of solid tumors, including TC (75, 76). According to research by Andreucci et al. (77), an acidic microenvironment increases the levels of melanoma cell stemness-related proteins (SOX2 and CAIX) and triggers an epithelial-mesenchymal transition (EMT) program. This increases the possibility that CSCs will self-renew and speeds up tumor growth. Conversely, Studies of solid cancers such as ovarian tumors and glioblastoma have demonstrated the important contribution of CSCs to the formation of an acidic tumor microenvironment (78, 79). For example, a study on ovarian tumors showed that CSCs exhibited higher rates of glycolysis, i.e., the ability of CSCs to uptake glucose as well as the amount of lactate and ATP produced were significantly elevated compared to ovarian cancer cells (78). Studies on PTC have confirmed that TCSCs contribute significantly to the formation of an acidic tumor microenvironment, as do thyroid cancer cells. For example, a study on PTCs showed that lactate levels in the growth medium of TCSCs were significantly elevated. This implies that TCSCs use a lactate efflux mechanism to help create an acidic tumor microenvironment (71).

### 3.2 TCSCs-immune cells crosstalk pathway

**Figure 4:**
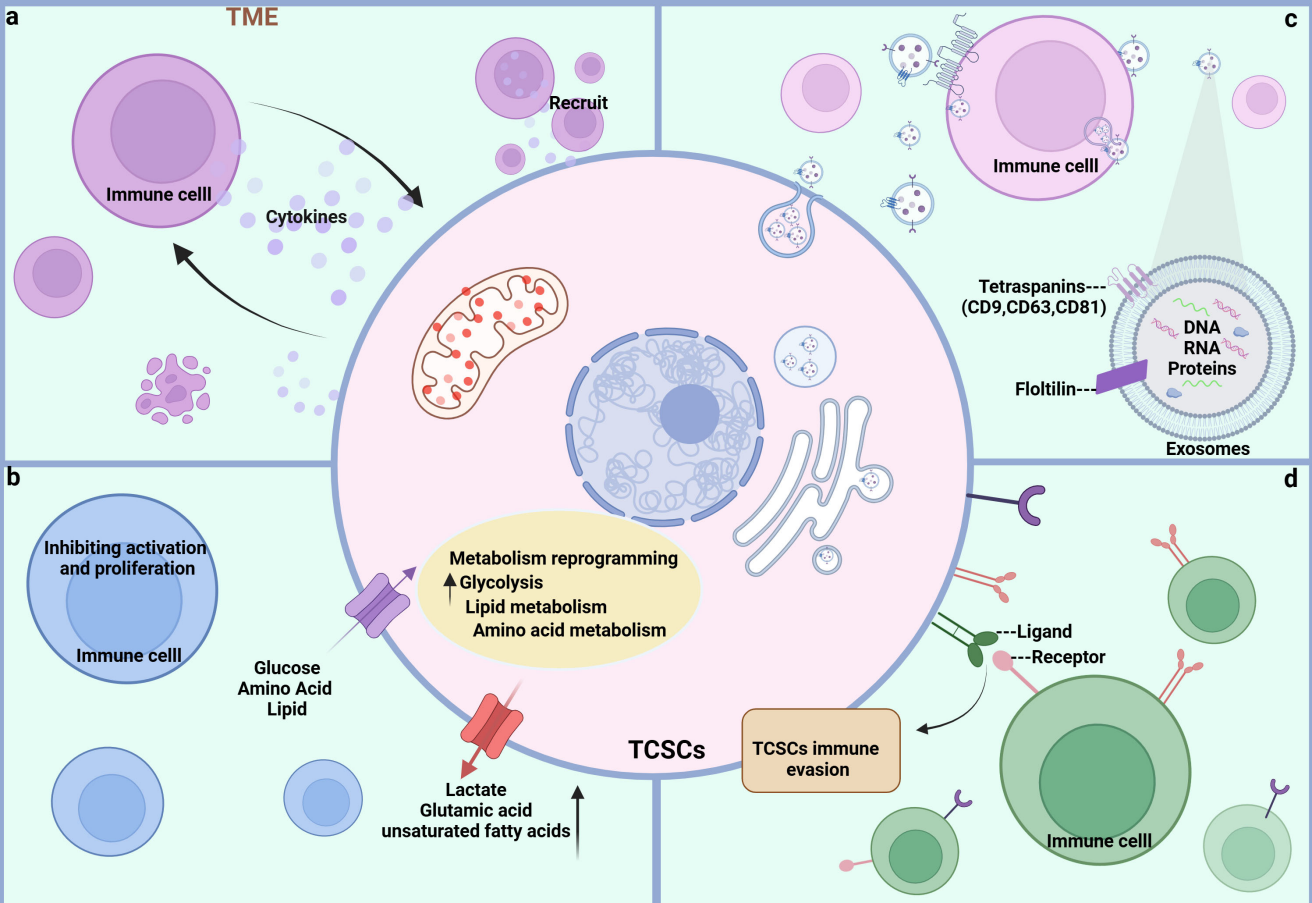
Crosstalk pathways between TCSCs and immune cells. **(A)** soluble molecules Both TCSCs and immune cells can release soluble molecules that facilitate information exchange and control one another’s reactions and behaviors, creating a dynamic web of interactions. **(B)** Metabolite Metabolites between TCSCs and immune cells can change each other’s phenotypic and functional states, further controlling their interactions. **(C)** EVs Immune cells and TCSCs can both release exosomes, which are microscopic vesicles that carry particular proteins and genetic information that are transferred from one cell to another to facilitate information sharing and function regulation. **(D)** Immune checkpoint TCSCs and immune cells recognize each other through ligands and receptors, forming signaling pathways that regulate immune responses and shape the immune microenvironment.

In the dynamic and complex TME, TCSCs exhibit highly complex bidirectional communication with various immune cells through multiple pathways. This ongoing, two-way biological process not only gives TCSCs the ability to avoid immune monitoring, but it also affects the phenotype and function of immune cells, which results in the development of an immunosuppressive milieu. In order to better understand the immunobiology of TCSCs and, consequently, to create more efficient therapeutic methods, it is necessary to comprehend the mechanisms by which TCSCs uniquely interact with immune cells. Here, we concentrate on the network of indirect communication that TCSCs have created with different immune cells in the TME via exosomes and different soluble substances and metabolites, as well as the pathway that directly expresses immune checkpoint molecules, such as PD-L1, to engage in an intimate dialogue with immune cells and induce an immunosuppressive microenvironment (**Figure 4**).

#### 3.2.1 Soluble material

In TME, soluble molecules (growth factors, chemokines, cytokines, and hormones, among others) released by CSCs and various immune cells are the basis for mediating the communication between CSCs and immune cells, which is essential for the intricate process of tumor evasion from immune surveillance and tumor progression because it not only controls the surface features of immune cells and their functional activity, but it also has a significant impact on the preservation of CSCs stemness and their capacity to induce carcinogenesis (80). First, TCSCs are able to secrete a series of immunomodulatory factors to recruit a variety of immune cells to the ecological habitat of the tumor. For example, chemokines generated by TCSCs, such as CCL2, CCL15, and CSF 1, can attract macrophages to the tumor ecotone (11), and more importantly, macrophages entering the ecotone of TCSCs can be polarized to M2-like TAMs by IL4, IL13, and TGF-ß to promote the formation of a tumor-immunosuppressive microenvironment (81, 82). In turn, immune cells can promote self-renewal, sphere formation, and tumor-initiating capacity of TCSCs by releasing molecular signals. For example, IL-8 released by mast cells maintains TCSCs stemness characteristics and tumor-initiating capacity through an Akt-Slug-dependent pathway (63).

#### 3.2.2 Metabolite

CSCs’ altered metabolism, such as higher glucose uptake and higher levels of lactic acid and unsaturated fatty acids, helps them meet their high energy needs while impairing the immune system’s capacity to recognize and eliminate them. It also changes the metabolism of immune cells, which encourages the growth of immune-suppressive cells and thereby impacts the TC’s advancement (83). Elevated lactate levels in TME have been shown to both directly or indirectly promote the acetylation of lysine 27 on histone H3 in B-cells and induce the differentiation of IL-10-producing regulatory B-cells, which can be used for immunosuppression to evade the host’s immune system, as well as impair the ability of natural killer (NK) to kill cancer cells by attracting myeloid-derived suppressor cells (MDSC) (84, 85). Along with reprogramming glucose metabolism, TCSCs have high glutamate levels, which support their fast growth and survival. Since glutamate is a crucial carbon source in the TCA cycle, high glutamate levels also reduce neutrophil cytotoxicity and stop T-cell proliferation (38, 72). To sum up, the metabolic interactions between TCSCs and immune cells cause nutrient depletion, immunomodulatory metabolite accumulation, and signaling pathway disruption in the TME. As a result, intervention strategies that target this metabolic interaction have promising anti-TCSCs and tumor-preventive effects.

#### 3.2.3 EVs

Recently, it has been recognized that there exists an emerging mechanism of interaction between CSCs and immune cells infiltrating the tumor microenvironment that involves the release of membrane-derived vesicles, known as extracellular vesicles (EVs) (86). EVs consist of a bilayer of phospholipid bilayers and double-layered membrane vesicles composed of soluble molecules, which serve as cellular mediators of intercellular communication “messengers” (87). Exosomes released by CSCs have been shown to carry non-coding RNAs in addition to biologically active molecules like proteins, DNA, and mRNA to surrounding and distant cells through receptor-ligand interactions, direct plasma membrane fusion, and phagocytosis, forming a bidirectional network of action, which in turn alters immune cell activity and tumor microenvironmental components, and affects tumorigenesis, growth, progression, metastasis, and drug resistance (88). It has been demonstrated that the exosomal lncRNA DOCK9-AS2, which is produced from TCSCs, activates the Wnt/β-catenin signaling pathway, increasing the stemness and tumor sphere-forming potential of thyroid cancer cells (89). Exosomes not only help TCSCs self-regulate, but they also alter immune cell phenotype and help TCSCs avoid the immunological response. For example, CSCs are able to release exosomes to regulate macrophage conversion to M2-like tumor-associated macrophage (TAM), inhibit T-lymphocyte proliferation and activation, and induce ecological niche of CSCs (90, 91). Conversely, MDSC-derived exosome (S100A9) can increase the stemness of cancer cells by upregulating genes linked to stemness (e.g., Oct4, Sox2, Nanog) in colorectal cancer cells and activating the NF-κB and STAT3 signaling pathways (92).

#### 3.2.4 Immune checkpoint

Immune checkpoints are important negative feedback mechanisms for maintaining the balance of the immune system under normal physiological conditions by limiting T-cell activity and avoiding over-activation of the immune system leading to tissue damage and a range of disorders in people (93). However, in thyroid malignancies, TCSCs can cleverly exploit a number of inhibitory immune checkpoints to avoid immune surveillance and encourage tumor growth (14). For example, a well-recognized immune checkpoint ligand widely expressed in tumor cells, PD-L1, transmits inhibitory signals when it is overexpressed on TCSCs and links to the receptor PD-1 on effector T cells, which is another important mechanism for crosstalk between TCSCs and immune cells (14, 94). The previously indicated interference mechanism deftly initiates the condition of T-cell depletion and inactivation in the pathological process of thyroid cancer, thereby evading the immune system’s vigilant watchful eye. This process is essential for controlling the development, metastasis, and recurrence of tumors, all of which have a significant impact on the course of the disease.

### 3.3 TCSCs-immune cell crosstalk

**Figure 5:**
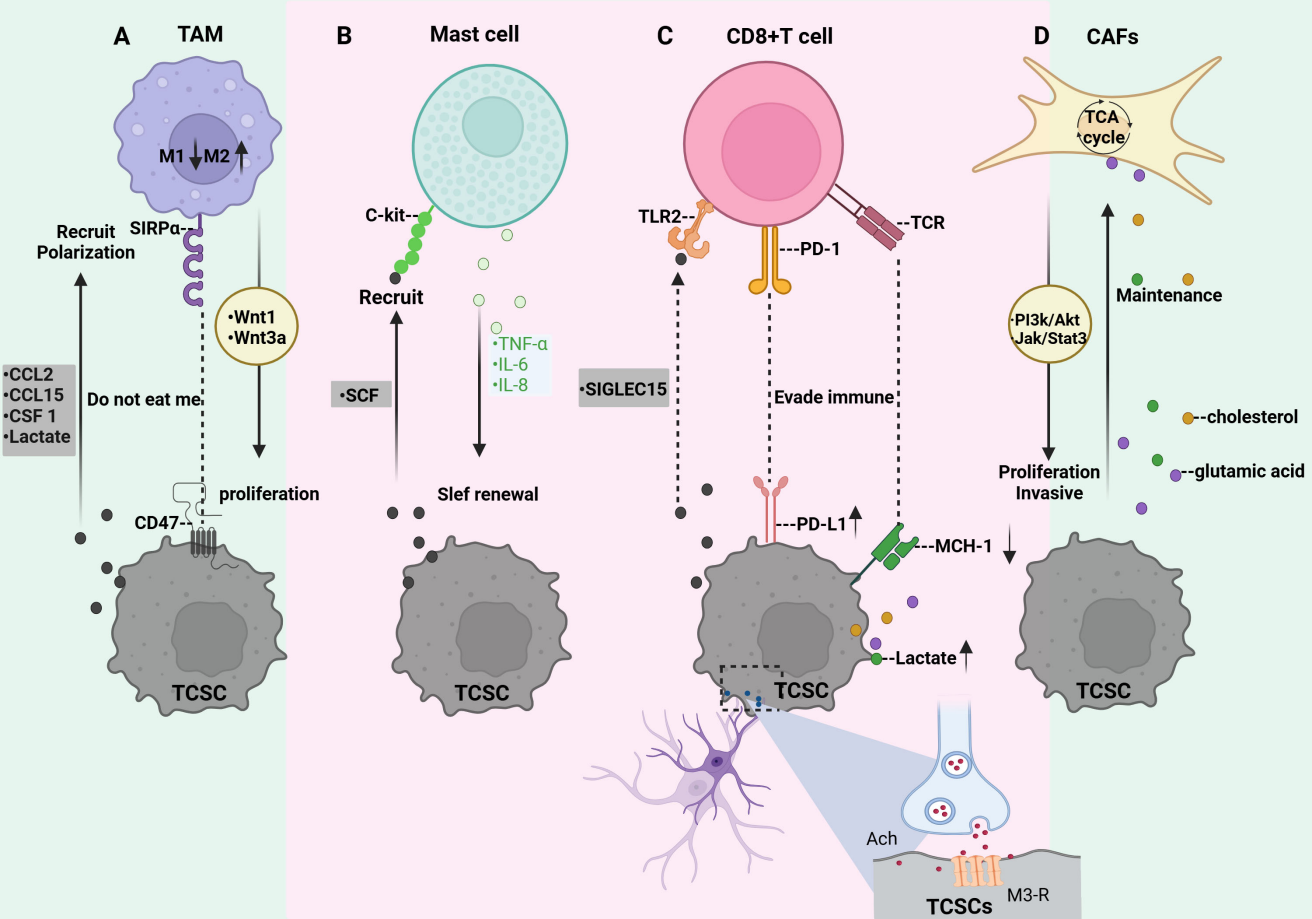
Crosstalk between TCSCs and immune cells. **(A)** TCSCs release immunomodulatory substances (CCL2, CCL15, and CSF 1) and metabolites (e.g., lactate) that promote macrophage recruitment and M2-like TAM polarization. TCSCs can also avoid being phagocytosed by macrophages by expressing CD47. TAM secretes wnt1 and wnt3a, which promote TCSCs’ ability to proliferate. **(B)** TCSCs release SCF to attract c-kit-expressing MCs into the TME, while MCs emit TNF-α, IL-6, and IL-8 to support TCSCs’ self-renewal ability, stem cell markers, and the EMT process for TC invasion and development. **(C)** TCSCs evade recognition and attack by CD8 + T lymphocytes either through immune checkpoints (MHC-I and PD-L1) or by secreting SIGLEC15. The acetylcholine produced by nerves acts on TCSCs to up-regulate the expression of PD-L1 and further inhibits the function of CD8+ T cells by competing for nutrients and the accumulation of metabolites, thereby promoting the self-renewal and immune escape of TCSCs. **(D)** Metabolites from TCSCs serve as substrates to increase CAF’s TCA cycling activity, which effectively sustains TCSCs’ high energy flux absorption. By regulating the JAK/STAT3 and PI3K/Akt signaling pathways, CAF encourages the growth and invasiveness of TCSCs.

As mentioned previously, TCSCs are located in the TME maintain their stemness state and plasticity, and possess immunological privileges that allow them to bypass both innate and adaptive immune regulation, thus ensuring their survival and development (95). By acting on immune cells through a variety of mechanisms, such as modified metabolic patterns to elude immune surveillance and release of immune cytokines and chemokines, the attraction and activation of inhibitory immune cells into the ecological niches of TCSCs, the up-regulation of immune-suppressing checkpoint ligands, and the selective enrichment of immune checkpoint receptors, TCSCs contribute to the formation of immunosuppressive TME (**Figure 5**). Therefore, to further understand this complex mechanism of TCSCs-immune cell crosstalk described above, we reviewed and summarized the current state-of-the-art insights into the crosstalk between TCSCs and immune cells, which will aid in the evolution of novel and more potent immunotherapies for the eradication of TCSCs.

#### 3.3.1 TCSCs-TAM

Tumor-associated macrophage (TAM), also known as macrophage, is one of the main innate immune cells in the TME, which influences tumor malignancy. Macrophages are usually converted to M1-like TAMs after the action of IFNγ, and TNFα, as well as lipopolysaccharides to exert tumor suppressive effects (96). When exposed to IL-4, IL-13, TGF-β, and other signals macrophages are induced to transform into a further class of M2-like TAM promoting tumor cell proliferation, stromal remodeling, neovascularization, and tumor niche modulation and interaction with CSCs contributes to the formation of immunosuppressive TME (81, 82). It has been demonstrated that more than 50% of immune cells in ATC are TAM (97). In the early stages of TC, M1 TAM are recruited and activate other immune cells, and as the disease progresses and the TME changes, M2-like TAM gradually increases (98), and the increased density of M2-like TAM is even more suggestive of an association with poor prognosis in thyroid cancer (99). Specifically, TCSCs recruit macrophages into the TME by releasing the immunomodulatory factors CCL2, CCL15, and CSF 1 (11) and contribute to the transformation of macrophages into M2-like TAM (11, 100). Additionally, the phenotypes of macrophages are influenced by metabolic mediators. For instance, it has been demonstrated that lactic acid generated from thyroid cancer cells (including TCSCs) stimulates the production of HIF-1α and activates the macrophage AKT/mTOR signaling pathway, increasing macrophage polarization towards the M2-like TAM (101). Importantly, TCSCs employ defense mechanisms to prevent macrophage phagocytosis in addition to polarizing macrophages into a protumorigenic state. CSCs can often evade macrophage immune control by overexpressing the “don’t eat me” signal CD47 (102). CD47 is a protein that can be detected in a variety of cancer kinds CSCs (CD47 is a transmembrane immunoglobulin widely expressed on CSCs of various tumor types, including LCSCs, PCSCs, and BCSCs (103, 104), and inhibits phagocytosis by macrophages by binding to SIRPα on the surface of macrophages, thus exerting the “don’t eat me” signal. effect, allowing CSCs to evade immune surveillance (105). In turn, TAMs may motivate the self-renewal and tumorigenic capacity of TCSCs, Using IL-4 and IL-13 cytokines, macrophages were successfully polarized towards M2-like tumor-associated macrophages (M2-like TAMs) in a co-culture experiment (106). After that, they were co-cultured with ATC cells, Tumor spheroids were greatly aided by this procedure, which also increased the expression of markers for stem cell characteristics such as Oct4, Sox2, and CD133. A thorough analysis showed that M2-like TAMs favorably regulated the IGF-1/IGF-2 signaling axis, which in turn prompted the activation of the PI3K/AKT/mTOR signaling pathway, activating the IR-A receptor and IGF-1R in ATC cells (106). This signaling pathway greatly expanded the invasive ability of ATC cells in addition to improving their stem cell-like characteristics. Recent research has demonstrated that TAM stimulates the proliferation and stemness of thyroid cancer cells by activating the Wnt/β-catenin signaling pathway through the release of Wnt1 and Wnt3a (107).

#### 3.3.2 TCSCs-Mast cells

Another subset of innate immune cells in the TME are mast cells (MCs). It has been reported that in addition to MCs being recruited into the TME by VEGF-A secreted by thyroid cancer cells (108), stem cell factor (SCF) released by thyroid cancer cells can also recruit MCs expressing CD117 (c-kit) into the TME (109). In general, in TC, MCs exert pro-tumorigenic and immunosuppressive responses by upregulating the expression of the immunosuppressive molecule galactoglucan lectin-9, which interacts with TIM-3 of Cytotoxic CD8 T cells (109). In addition, MCs produce a number of different mediators such as IL-6, IL-8, CCL25, CXCL10, CXCL, and TNF-α, which are engaged in TME remodeling and promote tumor development, metastasis, and neovascularization (110). Emerging evidence suggests that these mediators produced by MCs also perform a crucial function in the establishment and upkeep of TCSCs. Several studies have indicated that TC cells’ generation of reactive oxygen species or inflammatory signals can cause MCs to release TNF-α, IL-6, and IL-8, which in turn encourages the EMT process in thyroid cancer cells’ TC subtypes (FTC, ATC, and PTC) (63, 111); The creation of TCSCs may require the activation of EMT, a highly complicated cell biology process that controls cellular plasticity and activates a number of signaling pathways linked to stemness (28). For example, when FTC133 cells underwent EMT with HIF-1α, β-catenin underwent nuclear translocation, giving the cells extremely invasive and metastatic characteristics. Additionally, the number of stem-like SPs in thyroid cancer cells increased, demonstrating a greater capacity to create spheres and clones (112). Further studies revealed that IL-8 released from MCs mediates the EMT process and increases the stem cell-like features of thyroid cancer cells through a downstream Akt-Slug-dependent pathway, leading to TC progression (63, 111).

#### 3.3.3 TCSCs-CD8+T cells

CD8+ T cells, also known as cytotoxic T lymphocytes, serve as the main immune cell population for recognizing and eliminating cancer cells (113), which can be activated by recognizing tumor antigens presented by MHC class-I molecules. Activated CD8+ T cells migrate to the tumor region to release cytotoxic molecules such as IFNγ, TNFα, perforin, and granzyme B, which activate anti-tumor immune responses and induce apoptosis in tumor cells (114, 115). It has been revealed that CSCs can evade CD8 + T lymphocyte recognition and attack by down-regulating MHC-I, a process that significantly diminishes the sensitivity of CSCs to CD8+ T cell-mediated cytotoxicity, thus allowing CSCs to escape immune surveillance and potentially leading to tumor recurrence or metastasis at a later stage (116). In thyroid cancer, TCSCs were further found to interfere with CD8+ T cell activity by upregulating inhibitory immune checkpoint receptors on the surface with CD8+ T cells, which is an important pathway for them to escape immune surveillance (14). Interestingly, the nervous system within the tumor tissue is involved in this process and plays an immunomodulatory role. For example, a recent study on TCs demonstrated that nerves infiltrating TME act on TCSCs by secreting acetylcholine, causing them to activate the PI3K-Akt pathway, which in turn increases the expression of PD-L1 and thus enhances the resistance of TCSCs to CD8+ T cells (14). Notably, the up-regulation of PD-L1 expression also increased the expression of HK-2 and G6PD, two important CSCs regulators, which facilitated their pentose phosphate pathway and aerobic glycolysis processes, causing a significant reduction in glucose and buildup of lactate in the TME (117). This metabolic change not only hinders CD8+ T cells’ ability to function normally by depriving them of vital glucose, but the accumulated lactate can also further disrupt CD8+ T cells’ TCA cycle by inhibiting pyruvate carboxylase (PC), which reduces their anti-tumor activity (118, 119). By triggering the mevalonate metabolic pathway, CSCs also encourage the production and buildup of cholesterol. This deprives CD8+ T lymphocytes of their antitumor activities by encouraging them to decrease the expression of immunological checkpoints (120, 121). Furthermore, high cholesterol also sets off the endoplasmic reticulum (ER) stress response, which causes CD8+ T lymphocytes to become functionally exhausted. This leads to the growth and metastasis of tumors (122). According to published data, TCSCs that express high levels of Indoleamine 2,3-dioxygenase 1, a crucial enzyme in the metabolism of tryptophan, convert tryptophan to kynurenine, causing localized tryptophan depletion and significantly preventing the growth of T-lymphocytes stimulated by CD3 and CD28 working in concert (123). Emerging evidence points to the possibility that TCSCs may also evade CD8+ T cell clearance by expressing another immunosuppressive molecule, SIGLEC15 (124). SIGLEC15 belongs to the salivary acid-binding immunoglobulin-like lectin family gene family (125)and is an immunosuppressive factor produced by tumor-infiltrating myeloid cells as well as by specific tumor cells that is mutually exclusive with PD-L1 (126), and may bind to TLR2 and inhibit the activation of CD8+ T cells, suggesting that SIGLEC15-TLR2 interacts to transmit inhibitory signals and downregulate T cell immune responses (127). In co-culture experiments, SIGLEC15 was found to be highly expressed in ATC cells expressing the cancer stem cell marker CD44, SIGLEC15^high^ cancer cells not only express predominantly interact with T cells through immunosuppressive signals such as MIF-TNFRSF14 and CXCL12-CXCR4 to promote immunosuppression, but also reduce the production of anti-tumor cytokines IFN-γ and IL-2 by down-regulating the NF-κB/NFAT pathway, creating an immunosuppressive microenvironment conducive to tumor development (124).

#### 3.3.4 TCSCs-CAFs

Activated fibroblasts in TME are called CAFs, and they are a heterogeneous population of fibroblasts in TME that influence tumorigenesis, progression, and metastasis through the secretion of soluble factors, release of nutrients, remodeling of the extracellular matrix, and immunomodulatory functions (128). In a co-culture test on TC, soluble molecules including ROS and IL-6 released by undifferentiated thyroid cancer cells were discovered to transform fibroblasts into CAF via modulating the Src/Akt signaling pathway in fibroblasts (129). There are several ways that crosstalk between CAF and TCSCs supports the initiation and progression of TC, as evidenced by an expanding body of research. In order to maintain a high energy flux uptake by TCSCs, for instance, they increase the synthesis of lactate and glutamate through metabolic reprogramming. These metabolites act as sources of carbon and nitrogen, which encourage the growth of GLUL and genes encoding amino acid transferase enzymes that are in charge of glutamine synthesis in the CAF. This, in turn, increases the activity of TCA cycling in the CAF (38, 71, 130). Furthermore, CAF was shown to regulate the fate of TCSCs by mediating signaling. For example, Mirshahidi et al. (131) used TC-derived microenvironmental culture systems and patient-derived xenograft (PDX) models to explore and found that thyroid cancer stromal cells promoted TCSCs sphere formation, clonal proliferation, and invasiveness of TCSCs through mediating JAK/STAT3 and PI3K/Akt transduction of signaling in TCSCs. In addition, when interacting with CAF, increased Akt activity in TCSCs correlated with enhanced TCSCs invasiveness, and inhibition of Akt activity was able to reduce the tumorigenic ability of TCSCs (131). Inhibition of Akt signaling activity is therefore a potential therapeutic strategy for targeting TCSCs.

## 4 Targeting TCSCs

**Figure 6:**
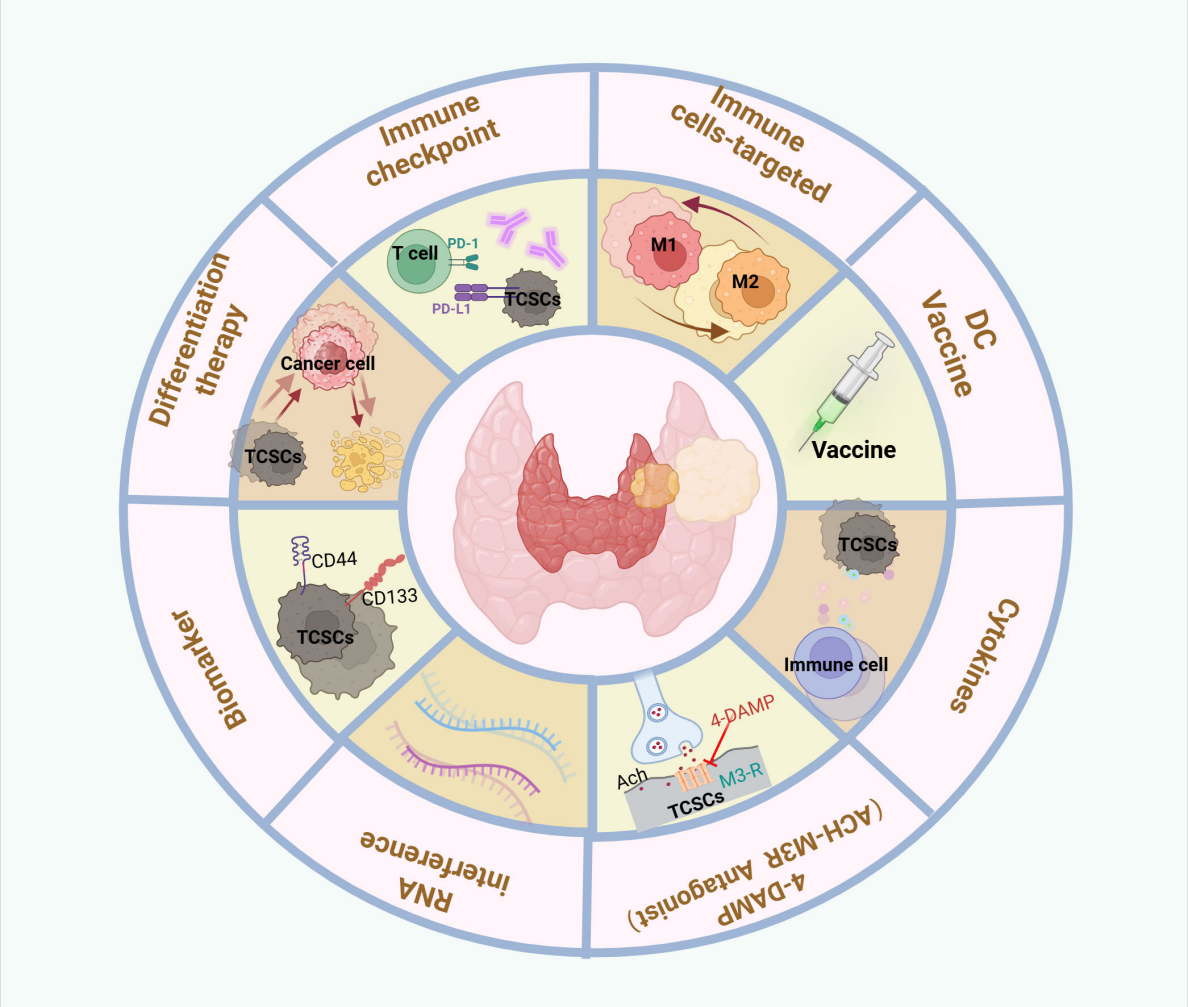
Therapeutic targeting of crosstalk between TCSCs and immune cells. Therapeutic strategies to target thyroid cancer stem cell and immune cell crosstalk may include the following: Targeting TCSCs, Immunotherapy, Combination therapy.

The molecular processes of TCSCs, such as stemness genes governing activity (e.g., CD133, CD44, ALDH, OCT4), aberrantly activated signaling pathways (e.g., Wnt/β-catenin, Shh, Notch), and metabolic vulnerability, have been intensively studied. These molecular mechanisms are crucial for the development, proliferation, invasiveness, and multidirectional differentiation potential of TCSCs, and their existence and abnormal activation are among the main causes of the resistance of TCSCs to conventional therapies. These not only complicate therapy, but also increase the risk of thyroid cancer metastases and recurrence. Eliminating TCSCs has been a primary objective in both fundamental research and therapeutic applications (132). Notably, in the realm of TC, new research has scrutinized TCSCs via significant genes, signaling networks, and metabolic processes; the findings are reviewed in this study (**Figure 6**).

### 4.1 Targeting biomarkers for TCSCs

With a deeper understanding of TCSCs biomarkers, significant progress has been made in developing targeted TCSCs. For example, De Andrade et al. (36) developed an AP-1-M-Dox coupling that combines the AP-1-M aptamer, which precisely recognizes and binds to the CD133 antigen, and the chemotherapeutic drug doxorubicin. In animal experiments, the AP-1-M-Dox coupling showed stronger anti-tumor ability and fewer toxic side effects than Dox alone, precisely targeting and killing TCSCs and inhibiting tumor growth, proving that CD133 is a potential target for the elimination of TCSCs Combined aptamer-mediated drug delivery improves the safety of treatment (36). In addition, Min et al. (133) found that PHGDH can not only participate in serine biosynthesis in TCs, but more importantly, can regulate the Sox2-Oct4 complex, a core stemness transcription factor that drives the progression and recurrence of TCs, and so may represent a possible target for thyroid cancer treatment. Nevertheless, given that the expression and types of surface markers of TCSCs may vary among different thyroid cancer subtypes and among different patients, therapeutic strategies in clinical studies should be tailored to personalize chemotherapeutic regimens based on phenotypic features unique to each cancer, so as to more precisely target and eradicate these heterogeneous TCSCs, and thus to improve therapeutic efficacy.

### 4.2 Targeting TCSCs-related signaling pathways

#### 4.2.1 WNT pathway inhibitor

GSK-LSD1, as a highly specific KDM1A inhibitor in the TCP family, successfully interfered with the activation of the Wnt/β-catenin signaling pathway through the inhibition of KDM1A, which significantly suppressed the ability of TCSCs formation of tumorspheres and the expression of TCSCs-related markers, OCT4, NANOGD, etc. expression (46). Given the excellent anti-tumor effects of GSK-LSD1, especially the significant inhibition of TCSCs, it shows great potential in the development of clinical therapeutic strategies targeting advanced thyroid cancer, heralding a new breakthrough in the field of precision medicine.

#### 4.2.2 NOTCH pathway inhibitor

Lee Doolittle et al. (50) showed that crenigacestat (An inhibitor of the NOTCH pathway γ-secretase) could block the formation of tumorspheres by inhibiting the NOTCH1-cMYC signaling axis, reduce aldehyde dehydrogenase activity, and effectively inhibit TCSCs-driven tumor initiation and development *in vivo*, in addition to revealing a novel Notch pathway inhibitory target-cyclin-dependent protein 7 (CDK7). Crenigacestat is being studied in a phase 1 clinical trial to treat advanced solid tumors (134). In a phase 1b study, crenigacestat, in combination with an anticancer drug, resulted in disease control rates of 62.5% (in combination with gemcitabine/cisplatin) and 60.0% (in combination with gemcitabine/carboplatin), with patients experiencing a treatment-emergent adverse event (TEAE) on at least one treatment. The majority of patients had ≥ grade 3 TEAE. the combination was therefore poorly tolerated, leading to clinically disappointing results in treating patients with advanced solid tumors (135). Clinical trials on Crenigacestat and other Notch inhibitors have been promising, but have achieved only modest efficacy, so there is a need to further explore the specific role and efficacy of Notch inhibitors for TC to ensure whether patients with TC will benefit.

#### 4.2.3 Hedgehog pathway inhibitor

Several studies have shown that the Shh pathway is overactivated in TC and is crucial for TCSCs self-renewal. Lu et al (136) investigated the role of the GLI-specific inhibitor, GANT61, in TC and found that GANT61 delayed the process of TCSCs-driven tumor growth by inhibiting the Shh pathway. According to the study, GANT61 shows promise as an inhibitor against putative TC therapeutic targets, opening up new avenues for the creation of treatment plans.

#### 4.2.4 JAK/STAT3 pathway inhibitor

The natural compound curcumin can target the JAK/STAT3 signaling pathway to induce cytotoxic effects in TCSCs (137). Specifically, curcumin, when paired with the chemotherapy agent cisplatin, more successfully inhibited the survival, and proliferation, and promoted the death of TCSCs by down-regulating the JAK/STAT3 signaling pathway; in addition, it suppressed the expression of stemness markers such as CD44, and ALDH, c-Myc, SOX-2, and Nanog (137). In addition, Cucurbitacin I, a STAT3 inhibitor, suppresses stemness gene signatures in TCSCs, upregulates the expression of thyroid-specific genes, and increases sensitivity to radiotherapy. Xenotransplantation experiments have shown that cucurbitacin I combined with radiochemotherapy significantly inhibits tumorigenesis and improves the survival rate of immunocompromised mice transplanted with TCSCs (138).

#### 4.2.5 PI3K/AKT-SOX2 pathway inhibitor

By inhibiting AKT, Diallyl trisulphide (DATS), a well-known hydrogen sulfide (H2S) donor, was able to restore the expression of thyroid-specific genes in ATC cells while decreasing the expression of the stemness marker SOX2 (139). This study implies that DATS can harm the expression of thyroid-specific genes in ATC cells by focusing on the AKT-SOX2 pathway. One potential intervention for the advancement of ATC is TCSCs (139).

### 4.3 Targeting the metabolic vulnerability of TCSCs

Numerous studies are focused on investigating novel therapies that target the mechanisms of metabolic interactions between TCSCs and immune cells at the metabolic level because these interactions are significant characteristic markers of TC that can affect TC progression and onset. For instance, it has been demonstrated that specifically targeting MTC4 in TC efficiently inhibits lactate release and increases the activity of PI3K/AKT and mTOR, key pathways in the proliferation of TCSCs (140). Through the knockdown of NOX1, Wang et al. (37) decreased the self-renewal and tumorigenic potential of TCSCs by inhibiting the activity of the PI3K/Akt pathway, which stimulates the formation of ROS. LW106 is a new small molecule IDO1 inhibitor that can decrease the percentage of CSCs and restore CD8+ T cell infiltration by preventing IDO1-mediated tryptophan depletion and metabolite buildup. This enhances the immune response in the tumor microenvironment and stops tumor growth (141).

Apart from focusing on important metabolic processes in TCSCs, the Dietary alteration of host metabolism has become a novel tactic in recent years, and in a mouse model of thyroid cancer, a ketogenic diet plus the antioxidant N-acetylcysteine effectively slowed the growth and progression of tumors (142). The metabolic changes of immune cells and the metabolic microenvironment of TCSCs should also be taken into consideration. Reversing the immunosuppressive metabolism milieu or boosting immune cells’ anticancer activity will help prevent TCSCs growth and proliferation and offer patients significant prognosis benefits.

### 4.4 Induction of TCSCs differentiation

Differentiation therapies aim to reprogram abnormally differentiated cancer cells to return to their normal path of growth and development, inducing these cells to mature and be eliminated by natural mechanisms (143). This fact has inspired differentiation therapies directly targeting immature CSCs (144), aiming to steer CSCs towards the final differentiated state of “normal” cancer cells, thereby eliminating their malignant features such as stemness. For example, according to Tseng et al. (138), ATC-CD133+ cells treated with cucurbitacin I were able to differentiate into ATC-CD133- cells, a process that was accompanied by notable alterations in biological characteristics. In particular, there was a notable decrease in their invasiveness and an attenuation of their capacity to form spheres and colonies. Furthermore, there was a notable upregulation of thyroid-specific gene expression, including important genes like NIS, TPO, and Tg. These modifications additionally facilitated the cells’ uptake of radioiodine. These findings strongly demonstrate the utility of cucurbitacin I as a differentiation inducer, especially in targeting TCSCs dependent on the STAT3 signaling pathway. Recently, the role of a new potential differentiation-inducing target, the transcription factor UHRF1, in inducing the differentiation of TCSCs was revealed through the application of mouse models and three-dimensional organoid culture techniques in mice and humans (145). It was observed that when UHRF1 was knocked down in thyroid cancer cells, not only markers of cellular dedifferentiation (e.g., CD97) were significantly decreased, but also stem cell-associated markers (Sox2, Oct4, and Nanog) were significantly reduced at both mRNA and protein levels, suggesting that down-regulation of UHRF1 expression could promote thyroid cancer cell differentiation at the transcriptional level, which is important for the containment of invasive thyroid cancer (ATC) development (145). Nevertheless, the application of differentiation therapy in thyroid cancer is still in the early stages of exploration, and more innovative approaches need to be developed to ensure that these findings can be effectively and precisely applied in clinical practice.

Despite the potential efficacy of certain targeted therapies against TCSCs, because of the intricate biology and flexible plasticity of TCSCs, and the intricate relationships that TCSCs have with various immune cell types that infiltrate the periphery. This poses certain challenges in identifying and targeting specific TCSCs populations for therapeutic strategies. First, treatments aimed at these targets cause damage to healthy tissue since many TCSCs target tumor-associated antigens rather than tumor-specific antigens. Second, it is extremely difficult to identify universal targets for TCSCs since they are a diverse collection of cells that express various markers, stemness genes, and functions rather than being a single cell population. Third, since TCSCs are very malleable and non-TCSCs can become TCSCs even after TCSCs have been eliminated, which can result in tumor recurrence. Therefore, targeting the TME that TCSCs survival depends on and overcoming their plasticity may be possible.

## 5 Immunotherapy targeting TCSCs

In recent years, a number of cutting-edge immunotherapeutic approaches, such as immune checkpoint inhibitors, macrophage reprogramming, and combination therapies, have emerged with fresh hope for overcoming these challenges (**Figure 6**). These strategies provide substantial prognosis advantages for patients by improving the immune system’s anti-tumor activity and the identification and destruction of TCSCs. Monoclonal antibody medications of the PD-1/PD-L1 class have been used to treat ATC and have shown promising clinical therapeutic outcomes (146).

### 5.1 Targeted Immune Cells

#### 5.1.1 TCSCs-TAM

M2-like TAM polarization to M1-like TAM would help to reverse immunosuppressive TME, activate the host immune system, and ultimately inhibit tumor progression. The FDA-approved anticancer drug bleomycin (BLM) is an antitumor antibiotic capable of inducing apoptosis by disrupting DNA in tumor cells, and it has been used primarily for the purpose of treating various cancer kinds (147–149). Using a low dose of BLM in a TC trial, it was found to not only selectively polarize M2-type macrophages to M1-type macrophages, but also promote apoptosis in thyroid cancer cells and effectively inhibit the progression of PTC (150), although high doses over a long period of time may cause severe pulmonary fibrosis (150). In addition, zoledronic acid (ZA), another drug that has been approved by the FDA for clinical use, may also inhibit the M2-type TAM polarization process, and it is an imidazole-containing bisphosphonate anticancer drug, which is generally used in TC for the treatment of patients with consolidation of bone metastases through the promotion of cell-cycle arrest, autophagy, and apoptotic (151–153). Using the colony and sphere formation experiment, Lu et al. (154) showed that ZA could prevent TAMs from polarizing toward the M2 type and impede the Wnt/β-catenin pathway-induced proliferation and stemness transition of TC cells. In addition, as previously described M2-like TAM-secreted IGF-1 and IGF-2 promote cancer invasion, stemness, and EMT in ATC through the IR-A/IGF-1R-mediated PI3K/AKT/mTOR signaling pathway (106). These findings shed light on the molecular pathways behind the progression of ATC, suggesting that another TAM-based treatment approach for TCSCs targeting could be the use of IGF1/IGF2 inhibitors (106).

#### 5.1.2 TCSCs-Mast cells

Mast cells trigger Akt phosphorylation and Slug expression in thyroid cancer cells by secreting the cytokine IL-8, which in turn maintains the EMT process and stemness properties of TC cells (63). Therefore, Visciano et al. (63) proposed that the EMT process of TC cells and their own stemness, and ultimately TC progression, could be reversed by inhibiting the Akt pathway or depleting Slug transcription factors. This study provides a new idea for further interfering with the downstream targets in the TCSCs-mast cell crosstalk pathway and thus treating TC, but further studies are needed to validate this.

#### 5.1.3 TCSCs-CD8+T cells

As mentioned previously, neuroreleased transmitters such as acetylcholine induce self-renewal of TCSCs as well as evasion of immunosurveillance by CD8+ T cells via the Akt pathway’s activation (14). Studies have demonstrated that acetylcholine receptor M3R antagonist (4-DAMP) effectively inhibits the growth of TCSCs *in vivo* and increases CD8+ T cells-mediated clearance of TCSCs (14). Therefore, targeting the acetylcholine receptor to inhibit the immune escape of TCSCs is a feasible therapeutic option to effectively kill TCSCs. In addition, Chimeric antigen receptor (CAR)-T cells involves modifying millions of exogenous T cells with chimeric antigen receptors and introducing them into the body to enable them to target specific antigens on CSCs (CSCs surface markers such as CD133, EpCAM, and ALDH) to recognize and kill CSCs (155, 156). Currently, the feasibility, safety, and preliminary efficacy of anti-CD133 CAR-T cells targeting CD133 in advanced malignant tumors (e.g., hepatocellular carcinoma) have been shown in phase I clinical trials (157). In addition, anti-EpCAM CAR-T cells demonstrated significant anti-tumor activity against CSCs of EpCAM solid tumors (e.g., colon cancer, breast cancer, etc.) without significant systemic toxicities (158). A CAR-EpCAM T-cells clinical trial (NCT03013712) is enrolling patients with EpCAM-positive cancers to evaluate the safety and efficacy of this therapy. Actually, CAR-T cells treatment aimed at TCSCs is also a promising immunotherapeutic strategy for TCs, but its lack of truly specific cell surface target antigens and the possibility of mistakenly killing normal cells remains unresolved (159), and further studies are needed to validate its safety and efficacy.

### 5.2 Targeted immune cytokines

Since TME has the prospective to support and initiate the transformation of thyroid cancer cells into TCSCs, targeting ecotropic factors that regulate the plasticity of TCSCs may be a more effective way of treating TC progression compared to directly targeting TCSCs. For example, IL-8 produced by immunosuppressive cells like cancer cells, mast cells, macrophages, and neutrophils (60–62) can promote TCSCs self-renewal, sphere formation, and tumor initiation capacity by attaching to TCSCs cell surface CXCR1 and CXCR2 via autocrine or paracrine pathways (62). Therefore, targeting the IL-8-CXCR1/CXCR2 axis could be used as an immunotherapy against TCSCs. Reparixin has been reported to be a variant, non-competitive, small molecule CXCL8 receptor (CXCR1/2) inhibitor (160). The effects of Reparixin on the function of TCSCs were assessed by RT-PCR, Flow Cytometry, sphere-formation and self-renewal, and the results showed that Reparixin (30 μM) significantly inhibited the self-renewal and sphere-formation ability of TCSCs; when it was combined with Docetaxel or Doxorubicin the tumor suppressive effect was enhanced (161). Thus Reparixin, either alone or in combination with classical chemotherapeutic agents, represents a potential therapeutic strategy for targeting key cytokines in the TCSCs-immune cell crosstalk process. The mechanisms by which TGF-β promotes thyroid cancer are multifaceted, and targeting TGF-β may be an effective approach to the treatment of TCs. Celastrol, which is isolated from the plant Thunder God vine a pentacyclic triterpene active ingredient capable of potent anti-tumor, anti-obesity, and anti-inflammatory activities (162). It has been found that Celastrol is effective in attenuating the TGF-β1-induced EMT process, stemness-related gene changes, and tumor migration in BRAFV600E cancer cells in TC (59). However, further trials are needed to demonstrate the reliability of Celastrol in inhibiting TCSCs in TC, and translating TGF-β-targeted therapies into clinical applications remains challenging.

The development of therapies that block pro-tumorigenic cytokines, like IL-8 and TGF-β, has shown that TCSCs can be prevented by modifying cytokines. As a result, we can try to increase the activity of interferons and interleukins (such as IL-2, IL-7, and IL-12, among others) that have both immunostimulatory and growth-inhibitory effects (163), which will provide more efficient treatment approach for the removal of TCSCs.

### 5.3 Targeted immune checkpoints

TCSCs overexpress a variety of immunosuppressive molecules such as PD-L1, SIGLEC15, and others to evade immune attack, so blocking suppressive immune checkpoints will reactivate or restore the killing function of anti-tumor-specific immune cells. Inhibition of the interaction between PD-1 and PD-L1 enhances the tumor-killing capacity of T cells, leading to possible antitumor effects (164). Currently, several phase II clinical trials targeting the PD-1-PD-L1 axis are underway in thyroid cancer, for example, a human phase II study demonstrated that Spartalizumab (PDR001 is a humanized immunoglobulin 4 monoclonal antibody), which binds to PD-1 and blocks the interaction between PD-1 and PD-L1 and PD-L2, showed promising results in advanced and metastatic ATC patients, demonstrating favorable clinical activity and a favorable safety profile (165). Pembrolizumab (as a PD-1 inhibitor) is currently being tested for efficacy in a Phase II clinical trial that focuses on patients with undifferentiated thyroid cancer for which there is no drastic alternative therapy available (NCT05119296). Concurrently, a second Phase II clinical trial is being conducted to investigate durvalumab’s (a PD-L1 inhibitor) potential for treating individuals with advanced thyroid cancer that is refractory (NCT03753919). In addition, another immunosuppressive molecule expressed in TCSCs, SIGLEC15, a member of the salivary acid-binding immunoglobulin-like lectin family gene family, modulates various immune cell-mediated immune responses, such as tumor-associated macrophages, CD8 T-cells, NK cells, and MDSCs (125). Bao et al. (124) applied anti-SIGLEC15 in TCs and found that it increased anti-tumor immune cells such as the ratio of M1/M2 and infiltration of CD8+ T cells and NK cells in the TME, and decrease the infiltration of pro-tumor immune cells such as M-MDSCs and (PMN)-MDSCs. In addition, anti-SIGLEC15 antibody induced IL-2 and IFN-γ secretion and T-cell activation through activation of the NF-κB/NFAT pathway (124). Overall, blocking SIGLEC15 not only inhibited TCSCs but also effectively reduced TC growth by reversing the immunosuppressive microenvironment. Recently, a phase I clinical trial of the first monoclonal antibody (NC318) targeting SIGLEC15 showed that patients with advanced/metastatic solid tumors with PD-L1 tumor proportion score (TPS) <50% and refractory or resistant to existing therapies could benefit from anti-SIGLEC15 treatment (NCT03665285). Therefore, specifically targeting SIGLEC15 with monoclonal antibodies could be a potential approach to intervene in TCSCs.

### 5.4 TCSCs-targeting novel immunotherapies

Several new immunotherapeutic approaches against CSCs are undergoing clinical trials, such as therapeutic cancer vaccines, which are a form of relay immunotherapy using antigen-presenting cells (APCs) to present tumor-associated antigens to specific T cells, thereby activating the T cells to eliminate the tumor cells (166). For example, the CSCs-DC vaccine significantly inhibited tumor recurrence and metastasis and prolonged animal survival after surgical resection in mice with SCC7 squamous cell carcinoma and D5 melanoma (167). In addition, oncolytic virus therapy (OVT), which acts against CSCs by inducing cancer cell death and activating anti-tumor cells, is also of interest (168). However, the physical barrier within the TME restricts viral dissemination to CSCs and may limit the efficacy of OVT. Currently, these novel immunotherapies in combination with standard chemotherapy have been shown to be feasible and effective in the treatment of CSCs, but also face significant challenges before they can be truly applied.

## 6 Conclusions and outlook

According to what was previously mentioned, TCSCs not only control the development of tumors through the regulation of stemness genes and cell-intrinsic factors like abnormally activated signaling pathways, but they also secrete immunosuppressive cytokines, attract and activate immune cells into the ecological niche that TCSCs occupy, and engage in complex communication with immune cells. Through these interactions, TCSCs preserve their stemness, oncogenicity, and resistance to treatment, as well as evade immune surveillance and clearance and modify the immunosuppressive tumor microenvironment, which ultimately promotes tumor progression.

In view of this, targeted therapies, differentiation therapies, and immunotherapies that target the crosstalk between TCSCs and immune cells have demonstrated great potential for eradicating TCSCs. Nonetheless, multiple challenges need to be overcome before the above strategies can be formally translated into practical clinical application protocols. First, the current stem cell markers for identifying and isolating TCSCs are almost exclusively those shared with normal stem cells, as well as the fact that individual stemness markers are unlikely to accurately characterize TCSCs subpopulations given the complex manifestations of plasticity and intra-tumor heterogeneity in TCSCs. Single-cell sequencing, in combination with spatial genomics technologies, offers promising avenues to accurately identify, isolate, and characterize specific TCSCs. Second, most experiments have investigated the characterization of TCSCs in ex vivo cellular or severely immunodeficient mouse models that lack a robust immune system, making it difficult to fully mimic the heterogeneity and complexity of the *in vivo* tumor microenvironment in humans. In recent years, researchers have used genetically engineered animal models (169) to mimic the pathophysiological features of cancer with relative accuracy, providing a platform for dissecting the cellular and noncellular components of the TME as well as investigating the crosstalk mechanisms between CSCs and the immune system. In addition, the latest organoid (170, 171) developmental techniques have significantly increased our ability to perform dynamic analysis of TMEs by simulating the three-dimensional (3D) structure of tumor tissues and complex TMEs to establish an *in vivo* physiological model that is more compatible with tumor progression than the 2D environment, complemented by lineage tracing to monitor the behavior of intact developing CSCs. Third, direct evidence on the mechanism of crosstalk between TCSCs and immune cells in the tumor microenvironment is still scarce, and the application of emerging technologies to thyroid cancer stem cells, such as the use of a combination of *in vivo* and real-time imaging to study TCSCs in living TCs, will help researchers to monitor the interaction between TCSCs and immune cells in real-time and explore new mechanisms of crosstalk between them as well as develop new strategies to overcome drug resistance. Fourth, studies have shown that TCSCs are plastic and dynamic populations that can change their cellular phenotype spontaneously or in response to certain stimuli such as viral infection, hypoxic stimuli, and induction by conventional therapies. One of the main challenges in focusing on TCSCs is their phenotypic plasticity and instability in TCs. Therefore, targeting TCSCs therapy alone is considered insufficient for effective tumor elimination. Combining TCSCs-targeted immunotherapy with conventional therapies (e.g., chemotherapy, radiotherapy, and checkpoint inhibitors) is a much more desirable strategy that would be expected to reduce drug resistance and achieve better efficacy. Fifth, despite the great success of immunotherapy in the treatment of many solid tumors, including thyroid cancer, immunotherapy targeting CSCs still suffers from a number of dilemmas, such as the difficulty in selecting CSCs-specific tumor antigens as highly efficient sites for recognition and binding in cancer vaccines and CAR-T cell therapies. Novel nanobiotechnology (172) can not only monitor the characteristics of CSCs more accurately and non-invasively but also significantly enhance the efficacy of targeted CSCs immunotherapy through the advantages of high-dose drug delivery and controllable drug release by using nanomaterials to bind specific CSCs surface markers and trigger signal amplification. Sixth, due to the dynamic changes and high complexity of TME, in addition to immune cells, the roles of other cells (e.g., neurons, adipocytes, and microorganisms, etc.) and non-cellular components in the ecological niche of TCSCs are also understudied and need to be explored in greater detail. Seventh, it is worth noting that tumor immunotherapy now faces the limitations of poor response rates and immune-related side effects, even if it is a state-of-the-art treatment. Through better drug delivery and fewer side effects, biomaterials have greatly increased anti-tumor effects in this context (173). They have also emerged as a crucial mediator in modifying TME and boosting immunotherapy’s effectiveness. Significant progress in the field of tumor immunotherapy is heralded by this interdisciplinary approach, and further research and use of this treatment are required.

In conclusion, an increasing amount of evidence suggests that TCSCs-immune cell interactions are key drivers of TC development, involving multiple cell signaling and transduction pathways, as well as various cell types and bioactive molecules. Interventional therapies targeting key targets and biological events during these interactions require rigorous validation of the targets and mechanisms behind them, and a deeper understanding of the surrounding tumor microenvironment in which they are embedded, which will greatly benefit thyroid cancer patients.
